# Hernia mesh infection treatment following the repair of abdominal wall hernias: A single-center experience

**DOI:** 10.3389/fsurg.2022.993855

**Published:** 2022-10-25

**Authors:** Linxiang He, Xuehu Wang, Gaoxiang Fan, Yu Zhao

**Affiliations:** The Department of Vascular Surgery, The First Affiliated Hospital of Chongqing Medical University, Chongqing, China

**Keywords:** abdominal wall hernia, mesh infection, laparoscopic exploration, open debridement, treatment

## Abstract

**Introduction:**

The mesh-based repair of abdominal wall hernias is a commonly employed approach as it is easy to implement and associated with low rates of hernia recurrence. However, the occurrence of hernia mesh infections following such repair can be extremely serious, and no clinical consensus regarding the optimal treatment of such infections has been established. This study was thus developed to review the management of hernia mesh infection cases treated at our center, summarizing the demographic and clinical characteristics of affected patients and summarizing our associated therapeutic experiences.

**Methods:**

Data pertaining to 64 cases of hernia mesh infections treated at our center were retrospectively reviewed. Data were obtained from patient medical records, including general situation, hernia type, prior hernia repair approaches, type of mesh, and postoperative condition. Other reviewed outcomes include bacteriological and imaging findings, as well as treatment outcomes. In cases where conservative management was not successful, the approach to mesh removal (laparoscopic vs. open) was made based on the primary surgical approach and the type of material used for the repair.

**Results:**

In total, 42 patients underwent primary open inguinal hernioplasty (including plug repair, preperitoneal mesh repair, and Lichtenstein repair), while 11 patients underwent laparoscopic repair (9 transabdominal preperitoneal, TAPP and 2 totally extraperitoneal,TEP), and 11 patients with incisional hernias underwent the intraperitoneal onlay mesh (IPOM) procedure. Six patients exhibited mesh erosion of the internal organs. Of these patients, 38 underwent mesh removal *via* open debridement, while 9 underwent laparoscopic exploration and open debridement, and 1 underwent laparoscopic mesh removal. No patients exhibited serious postoperative sequelae, serious complications, or mortality after the treatment of mesh infections.One patient experienced postoperative infection recurrence following partial mesh removal, with the appearance of a small fistula. Hernias recurred in 2 patients following mesh removal, and 1 patient underwent repair *via* laparoscopic IPOM.

**Conclusions:**

While conservative treatment can cure early mesh infections, there is nonetheless a risk that these infections will recur. In view of the variety of surgical intervention of abdominal wall hernias at present,treatment of mesh infection should be individualized. Our findings suggest that hernias repaired *via* the placement of mesh in the preperitoneal space can more readily contribute to internal organ erosion and late-onset infections, with open debridement often being unable to completely remove the mesh without causing collateral damage. Laparoscopic exploration is an effective and minimally invasive approach to detecting internal organ involvement and removing the infected hernia mesh from affected patients.

## Introduction

Abdominal wall hernias are a common clinical entity, affecting 1.7% of the general population and 4% of individuals over 45 years of age (1). Roughly 75% of all abdominal wall hernias are inguinal hernias, which are associated with a lifetime risk of just 3% in women but 27% in men (2). As suture-based hernia repair is associated with very high rates of postoperative recurrence, the use of a reinforcing mesh has emerged as a common reparative procedure that is widely accepted and employed in the treatment of abdominal wall hernias.

While mesh-based hernia repair is associated with significant reductions in hernia recurrence rates, this procedure is associated with certain risks. The most severe of these is the potential for the development of a hernia mesh infection, which can lead to significant increases in patient morbidity and healthcare costs (3). Recent estimates suggest that postoperative mesh infection rates may be as high as 2.2% in ventral hernia patients (4), rising to as high as 7.3% for patients that undergo open sublay ventral hernia repair, while laparoscopic and open mesh repair procedures have variously been reported to exhibit mesh infection rates of 3.6% and 10%, respectively (5, 6).

Several approaches to treating mesh infections have been reported to date, but no standard treatment strategy is applicable to all affected patients. There is thus a clear need for the individualized optimization of mesh infection treatment. This study was thus developed to review our single-center experience in the management of mesh infection cases so as to analyze the demographic and clinical characteristics of affected patients and to provide a summary of our experiences.

## Materials and methods

This was a single-center retrospective analysis. Patients included in this analysis were those that developed hernia mesh infections following the repair of incisional or ventral hernias. Patients were excluded if they developed superficial surgical site infections that did not extend to the mesh. In total, data from 64 mesh infection cases treated at our center from February 2012–September 2021 were reviewed. Among them, 58 cases were transferred from other hospitals. Most of the patients primarily underwent either unsuccessful conservative treatment or incomplete mesh excision prior to referral to our department. All patients provided informed consent.

**Figure 1:**
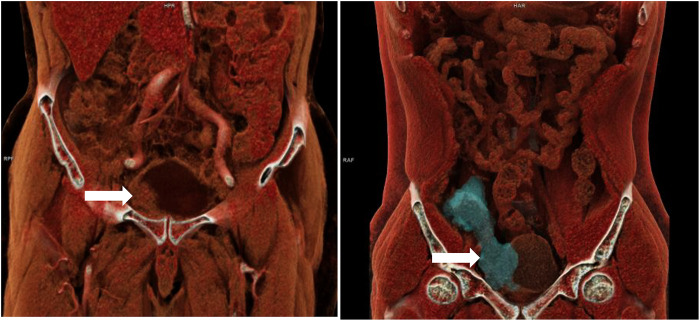
Three dimensional reconstructive CT of two patients with bladder invasion after right inguinal hernia repair.

**Figure 2:**
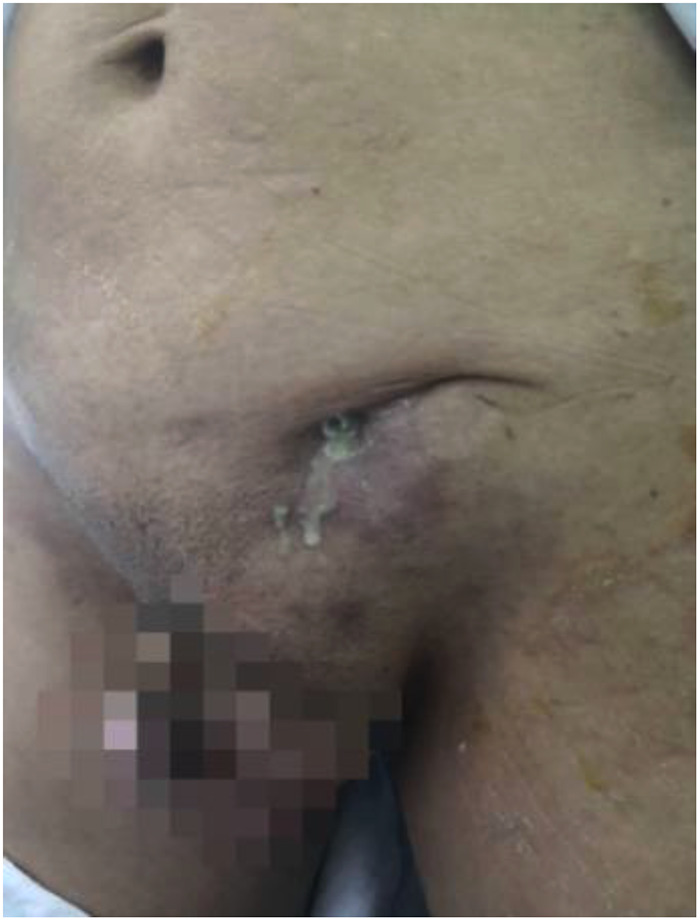
A patient with intermittent discharge after left inguinal hernia repair 2 years later.

All patients underwent preoperative contrast-enhanced computed tomography (CECT) scans Figure 1. In patients where internal organ invasion was suspected (bladder, colon, small intestine, vessels), cystoscopy, enteroscopy, and sinus contrast radiography were performed. Discharge from the infected mesh or abdominal wall sinus was collected for culture and antibiotic sensitivity testing Figure 2. The patient management strategy used in this study is outlined in Table 2.

**Figure 3:**
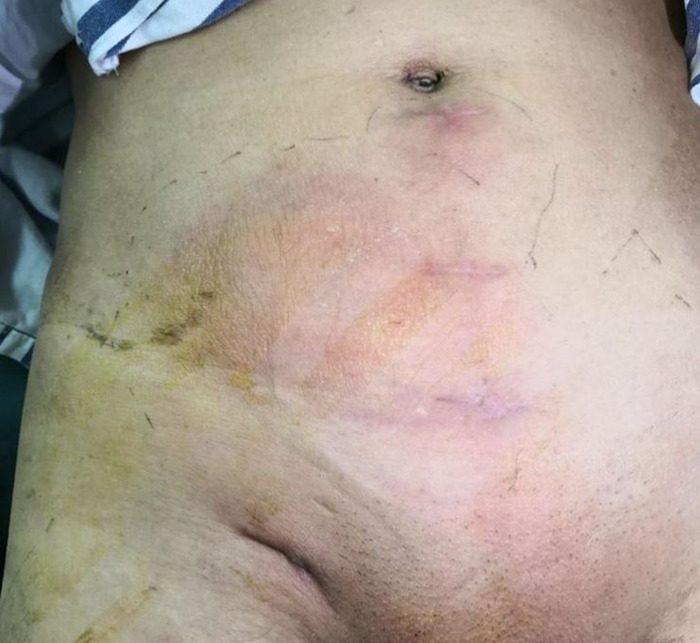
A patient with local swelling, erythema and fever after right inguinal hernia repair (TAPP) 2 weeks later.

Patients with acute infections (especially including those that underwent Lichtenstein or IPOM repair, or repair with biological patches) were treated with intravenous antibiotics, percutaneous drainage, and negative pressure wound therapy Figure 3. Mesh removal was performed for those patients exhibiting poor responses to conservative treatment for more than one month.

The operative approach for mesh removal (laparoscopic vs. open) was selected based on the primary surgical approach employed and whether or not invasion of the internal organs was observed. For patients that had initially undergone Lichtenstein repair, the infection mesh, sinus, and affected tissues were excised *via* an anterior approach. For patients that had undergone laparoscopic/open preperitoneal/plug hernioplasty, laparoscopic examinations were performed following bowel preparation. This laparoscopic approach was used to fully explore the pelvic cavity to detect any adhesions and to localize infection foci. Any adherent bowel loops or omentum were gently separated from the inguinal region. To explore Retzius' space in cases of preperitoneal mesh or plug erosion of the bladder, the peritoneum was incised while avoiding contamination of the abdominal cavity. Simultaneous bowel repair was performed when appropriate.

**Figure 4:**
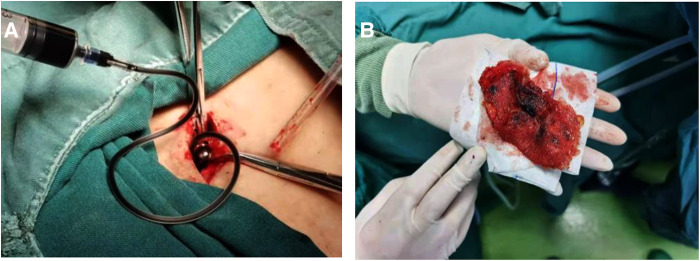
Surgical procedure of infected mesh removal using methylene blue. (**A**) Methylene blue was injected in sinus on incision site. (**B**) Infected Kugel patch was isolated and removed.

When laparoscopic exploration yielded no positive findings or internal organ invasion was detected, the decision was made to convert to an open surgical procedure. Initially, a shuttle-shaped incision including the sinus was made, after which the sinus tract, complete mesh, non-absorbable sutures of glue, and contaminated tissues were removed from the affected site based on the results of methylene blue staining Figure 4.

**Figure 5:**
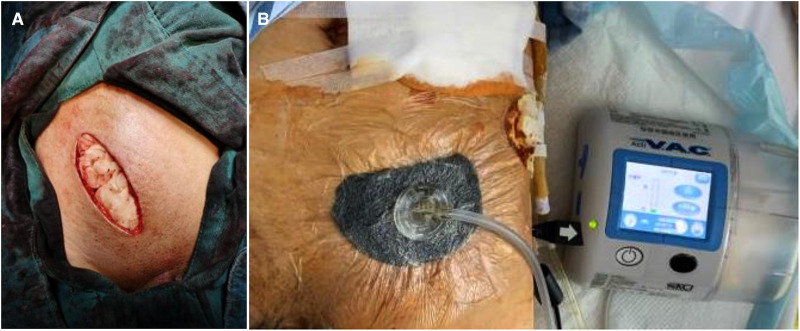
(**A**) the wound was filled with gauze when operation ended; (**B**) the negative pressure wound therapy was applied.

After the wound was cleaned as much as possible, it was closed with vacuum drainage. Wounds with large abscess cavities or severe infections were filled with gauze, and negative pressure wound therapy was applied following the cessation of bleeding, with secondary suturing being performed after the wound condition had improved Figure 5.

Intravenous antibiotic administration was performed based on the results of culture and sensitivity testing. Ultrasonography or computed tomography (CT) scans were used to assess wound healing.

All statistical analyses were performed using SPSS 26.0.

## Results

In total, data pertaining to 64 patients (55 male, 9 female) were included in this study. These patients had an average age of 57.8 years, and an average body mass index (BMI) of 24.3 kg/m^2^ (Table 1).

**Table 1:** Baseline patient characteristics.

	Value
Age (years)	57.8 ± 12.5
BMI (kg/m^2^)	24.3 ± 1.7
Comorbidities [*n* (%)]	
Diabetes mellitus	5 (7.8%)
Current smoker	36 (63.8%)
History of malignant tumors	3 (4.6%)
Chronic kidney disease	2 (3.1%)
Hypertension	7 (10.9%)
Primary hernia [*n* (%)]	
Inguinal hernia	40 (62.5%)
Recurrent inguinal hernia	8 (12.5%)
Bilateral inguinal hernia	5 (7.8%)
Incisional Hernia	11 (17%)
Primary hernioplasty method [*n* (%)]	
TAPP approach	9 (14.1%)
TEP approach	2 (3.1%)
Open preperitoneal approach	2 (3.1%)
Lichtenstein	24 (37.5%)
Plug repair	16 (25%)
IPOM	11 (17.2%)
Type of infected mesh [*n* (%)]	
PPE	60 (93.7%)
Biological meshes	4 (6.3%)
Mean time to infection diagnosis (months)	19.4
Symptoms [*n* (%)]	
Abscess	4 (6.3%)
Chronic sinus infection	40 (62.5%)
Local swelling and erythema	19 (29.7%)
Fever	4 (6.3%)
Debridement history [*n* (%)]	38 (59.4%)
Debridement ≥2	6 (25%)

BMI, body mass index, TAPP, transabdominal preperitoneal prosthesis, TEP, totally extraperitoneal prosthesis, IPOM, intraperitoneal onlay mesh, PPE, polypropylene. The symptoms of abscess and fever were combined in 3 patients.

In total, 53 patients (82.8%) exhibited comorbidities prior to undergoing the primary procedure, including diabetes mellitus (7.8%), current smoker status (63.8%), a history of malignant tumors (4.6%), hypertension (10.9%), and chronic kidney disease (3.1%). Moreover, 8 patients (12.5%) presented with recurrent inguinal hernias, while 5 (7.8%) were affected by bilateral inguinal hernias.

The results of bacterial culture and sensitivity testing are summarized in Table 2. Of the included patients, 3 exhibited MRSA infections, while 8 were affected by mixed bacterial infections.

**Table 2:** Patient bacteriological culture results.

Organism	*n* (%)
Gram-positive	30 (47%)
Staphylococcus spp.	22 (34%)
Staphylococcus epidermidis	4 (6%)
Enterococcus faecalis	3 (5%)
Corynebacterium xerosis	1 (1%)
Gram-negative	19 (30%)
Escherichia coli	7 (11%)
Pseudomonas spp.	11 (17%)
Klebsiella pneumoniae	1 (1%)
Candida albicans	1 (1%)
Not detected	14 (22%)

Primary procedures performed in 42 of these patients were open inguinal hernioplasty operations performed using techniques including plug repair (*n* = 16), preperitoneal mesh repair (*n* = 2), and Lichtenstein repair (*n* = 24). Laparoscopic approaches were used to treat 11 patients, including TAPP repair and TEP repair procedures, while 11 patients with incisional hernias underwent IPOM procedures, of whom 3 experienced mesh removal following secondary repair, and 1 underwent a simultaneous mesh repair procedure following *en bloc* resection. PPE was applied in 60 patients (93.7%), while biological meshes were used in 4 patients (6.3%). Of the included patients, 38 (59.4%) patients had a history of debridement surgery, of whom 16 (25%) had undergone more than two debridement surgery procedures.

The average interval between initial hernia surgery and infection diagnosis was 19.4 months (range: 1 week–12 years), with 41 patients being diagnosed in <3 months, 14 patients being diagnosed between 3 months and 1 year after the primary procedure, and 19 patients being diagnosed >1 year after the primary procedure. Patient symptoms included abscess, chronic sinus infection, localized swelling, fever, and erythema. Laboratory testing in most patients was consistent with an elevated inflammatory index (C-reactive protein and white blood cell counts). CECT examinations revealed extensive infections in the original operative site.

Of these patients, 48 (75%) exhibited acute infections less than 1 month following hernioplasty that became chronic when not effectively treated. In one patient, after initial wound healing, infection recurrence was observed after 3 years. Sixteen patients (25%) exhibited late-onset mesh infections occurring from 2 months to 12 years following hernioplasty, with 10 of these patients having undergone laparoscopic/open preperitoneal/plug repair procedures through which mesh was placed in the preperitoneal space.

**Figure 6:**
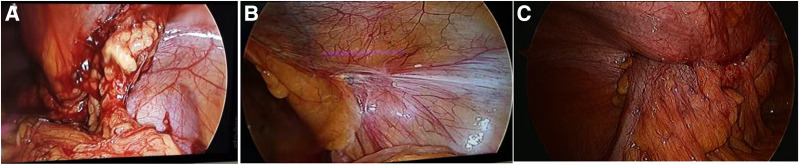
The mesh infection in laparoscopic exploration: (**A**) open preperitoneal hernioplasty showed adhesion of greater omentum; (**B**) lichtenstein repair showed no adhesion of inguinal region; (**C**) TAPP showed invasion of colon.

Patient treatment is detailed in Table 3. Of these patients, 16 (25%) were cured *via* conservative treatment (including intravenous antibiotics, percutaneous drainage, and negative pressure wound therapy), while 38 (60%) underwent open debridement-based mesh removal, and 9 (14%) underwent laparoscopic exploration and open debridement Figure 6. One patient (1.6%) that had undergone repair with a bovine pericardial patch *via* TEP underwent laparoscopic mesh removal. Six patients exhibited mesh erosion of internal organs, including 4 patients that underwent mesh-plug repair and 2 that underwent TAPP. Three patients exhibited mesh erosion of the colon, and following mesh removal a simultaneous bowel repair procedure was performed. Of these three patients, 1 underwent colon resection and anastomosis. Bladder erosion was evident in two patients, with hematuria as the presenting symptom in one of these patients. Iliac vein erosion was observed in one patient, resulting in a high degree of intraoperative bleeding (400 ml). Eleven patients (17%) underwent simultaneous wound suturing, while the wound was filled with gauze in 53 patients (83%).

**Table 3:** Patient evaluation and treatment.

Preoperative examination [*n* (%)]	
Computed tomography	64 (100%)
Cystoscopy	2 (3.1%)
Colonoscopy	3 (4.7%)
Treatment [*n* (%)]	
Conservative treatment (Intravenous antibiotics, percutaneous drainage, and NPWT)	16 (25%)
Open debridement	38 (60%)
Bowel resection and anastomosis	1 (1.6%)
Repair of colon	1 (1.6%)
Repair of bladder	2 (3.2%)
Repair of iliac vein	1 (1.6%)
Laparoscopic exploration and open debridement	9 (14%)
Repair of colon	1 (1.6%)
Laparoscopic debridement	1 (1.6%)
Wound management [*n* (%)]	
Primary suture	11 (17%)
Filled with gauze	53 (83%)
Mean time of operation (min)	79
Mean postoperative length of stay (days)	29

NPWT, negative pressure wound therapy.

Operative duration values ranged from 60 to 360 min, with blood loss ranging from 50 to 400 ml. The mean postoperative duration of hospitalization was 29 days. No patients experienced serious postoperative complications, sequelae, or mortality.

Follow-up duration for included patients ranged from 6 to 108 months, with infections recurrent in 1 patient that had undergone partial mesh removal appearing in the form of a small fistula. Inguinal hernia recurrence was observed in 2 patients (3.1%) at 9–12 months after mesh removal, with 1 of these patients undergoing a second hernioplasty *via* the laparoscopic IPOM approach, revealing residual mesh in Bogros' space.

## Discussion

Mesh infections following hernia repair are a cause of significant morbidity, resulting in hospital readmission, reoperation, and hernia recurrence, all of which contribute to the increased utilization of healthcare resources (7). Despite extensive efforts to maintain aseptic conditions during hernia repair procedures and through proper preoperative preparation, a limited subset of patients nonetheless develop mesh infections.

Previous work suggests that patient age, steroid use, obesity status, history of smoking, type 2 diabetes mellitus status, american society of anesthesiologists (ASA) score III/IV, laparotomy, emergency surgery, operative duration, and onlay mesh positioning are all significant risk factors associated with the odds of mesh infection incidence (8). We observed similar findings, and also determined that recurrent hernias and bilateral inguinal hernias may represent risk factors associated with mesh infection incidence owing to the consequent prolongation of operative procedures.

The degree to which prophylactic antibiotic administration can protect against mesh infections following hernia repair remains a subject of debate, with the dominant view being that such treatment is typically unnecessary. Nonetheless, some studies have reported benefits to prophylactic antibiotic treatment before open mesh hernia repair (9). Gram-positive bacteria are the primary cause of mesh infections, with *Staphylococcus aureus* as the most common causative pathogen, with methicillin-susceptible *S.aureus* (MSSA) being more common than methicillin-resistant *S. aureus* (MRSA), and some cases being caused by gram-negative bacteria such as *Escherichia coli* and *Pseudomonas aeruginosa* (10). In this study cohort, just 3 patients developed MRSA infections, suggesting that routine antibiotic treatment may benefit individuals at a high risk of developing mesh infections. The colonization of surgical implants by bacteria does not guarantee subsequent infection, with most colonized implants ultimately not becoming infected (11).We also observed negative culture results in some patients, likely because certain pathogens are difficult to detect through conventional culture, indicating that high-throughput sequencing strategies may enable more reliable pathogen detection and more effective treatment.

Most patients develop mesh infections weeks to months after hernia repair, and only very rarely exhibit a delayed infection after years have passed (12). This suggests that some of the patients in the present study exhibited a prolonged asymptomatic infection period following initial hernia repair or the curing of initial infections. Bacterial biofilms can reduce effective mesh porosity, thereby causing non-suppurative mesh-related complications while also potentially contributing to late-onset suppurative infections (13). Undetected biofilms composed of *Staphylococci* can contribute to these late-onset infections (14), potentially explaining the failure of conservative treatment measured and the delayed onset of infection for some patients in this study.

During the early stages of mesh infection, symptoms are atypical and may include local swelling or incision rupture in patients that have undergone laparotomy hernioplasty, while fevers, abscess formation, and erythema are more common in patients that undergo laparoscopic surgery. This may be due to the positioning of mesh in these patients, as superficial infections are less likely to result in systemic symptoms, while deep infections can be more damaging. CT is the most effective means of detecting infection development following mesh repair for patients with acutely incarcerated ventral and groin hernias (15). In our experience, CECT was an effective means of guiding debridement by clearing revealing infectious foci and nearby organs.

The most critical clinical goals when treating mesh infections are curing the initial infection and preventing the recurrence of that infection or the associated hernia. Optimal treatment strategies for mesh infections, however, remain uncertain as there have been few large-scale studies of patients with such infections, nor are there any evidence-based guidelines regarding the optimal timing of mesh removal in patients with persistent infections. As conservative treatment is only likely to result in the temporary abatement of infection and a prolonged treatment process, most affected patients ultimately need to undergo complete mesh removal (16). Most patients included in our study had undergone unsuccessful debridement procedures due to incomplete mesh or fixed object removal.

The mesh removal strategy employed is dependent on the method and material used for primary hernioplasty (17). In this study, some patients with acute infections were cured through conservative treatment (antibiotics or percutaneous drainage), although in some patients these infections recurred. As the peritoneum is resistant to infection, most patients that underwent IPOM repair procedures could be cured through conservative measures, whereas the lack of drainage resulted in the formation of an abscess in patients that underwent TAPP/TEP repair. When conservative treatment fails, mesh removal remains the treatment of choice. PPE undergoes measurable changes in its mechanical properties, crystallinity, and surface chemistry related to mesh placement, class, and infection (18). In our experience, mesh degeneration resulted in some amount of mesh remaining following open mesh removal. Conservative treatment can often resolve absorbable biological patch infections, and methylene blue injection can aid in maximizing mesh removal when treating patients suffering from mesh infections (19). We previously employed a laparotomy-mediated anterior approach to mesh removal, resulting in a large wound and the need for constant dressing changes. The advancement of laparoscopic procedures, however, has enabled us to probe behind the deep inguinal ring in a less invasive manner. The urogenital fascia ensheaths the urinary bladder in the inguinal region, supporting the identification of the appropriate anatomical level during TAPP/TEP procedures to reduce mesh erosion into the bladder (20). In most cases, laparoscopic exploration can effectively identify the foci associated with a mesh infection and assess whether or not internal organs are affected while minimizing any collateral damage (21). These exploratory laparoscopic approaches were most beneficial in patients in whom the hernia mesh had been placed in the preperitoneal space, whereas we found them to be less useful in patients that had undergone Lichtenstein repair, instead potentially resulting in increased damage. As such, an individualized approach should be used to select the mesh removal strategy based on previous repair operation details.

The most dangerous complication associated with mesh infections is the mesh invasion of internal organs, which can be potentially life-threatening. PPE can, in some cases, elicit chronic inflammatory responses resulting in scar tissue deposition and the formation of a dense layer of fibrous tissue around the mesh material, potentially resulting in bowel obstruction, chronic discomfort, infertility, perforation, or enterocutaneous fistula formation (22). We found that hernia repair procedures wherein the mesh was placed in the preperitoneal space were more likely to result in internal organ invasion. The causal relationship between such invasion and infections remains poorly understood. In our daily laparoscopic herniorrhaphy practice, we found sliding inguinal hernias (colon or bladder) to be common, and these may represent a risk factor associated with mesh invasion of the internal organs following TAPP/TEP repair procedures.

Postoperative NPWT application to closed incisions following VHR has been reported to reduce rates of hernia recurrence and wound complications (23).We observed low hernia recurrence rates in inguinal hernia patients following mesh removal. One possible explanation for this finding may be that extensive inflammatory cell infiltration following mesh infection may stimulate the infiltration and proliferation of fibroblasts, strengthening the transverse fascia. Alternatively, early hernia recurrence may not present with any symptoms, instead only being detected *via* imaging analysis.

## Conclusions

This was a retrospective analysis of a limited number of patients from a single-center, and future large-scale randomized controlled trials with a longer follow-up duration will be critical to more fully explore postoperative outcomes including hernia and infection recurrence. Even so, our findings suggest that early mesh infections can be cured through conservative treatment, but often progress to cause chronic or late-onset infections. These late-onset mesh infections are serious hernioplasty-related complications. In our experience, laparoscopic exploratory procedures can effectively detect internal organ involvement through a minimally invasive approach while supporting the removal of the infected mesh. In this patient cohort, we found that mesh infections after Lichtenstein repair had no intra-abdominal adhesions and no positive findings in the groin area through a laparoscopic exploratory. To sum up our experience, once the mesh infection is diagnosed, conservative treatment should be controlled within two weeks. For mesh infection after Lichtenstein repair, open debridement is needed. For mesh infection after laparoscopic/open preperitoneal/plug hernioplasty, and laparoscopic examination are needed, Laparoscopic mesh removal can be considered if necessary. The observation that hernia recurrence rates were low following mesh removal in individuals that had developed inguinal hernia infections is an interesting finding, but further study will be needed to clarify the mechanisms underlying this observation.

## Data Availability

The original contributions presented in the study are included in the article/Supplementary Material, further inquiries can be directed to the corresponding author/s.
